# Supplementary material for: Phenotypic Assessment of Pathogenic Variants in GNAO1 and Response to Caffeine in C. elegans Models of the Disease
Source: Genes (Basel). 2023 Jan 26;14(2):319. doi: 10.3390/genes14020319 (PMC9957173; doi:10.3390/genes14020319)
Supplement: Supplementary file 1 [file genes-14-00319-s001.zip › genes-2141421-supplementary.pdf]

Supplementary Materials

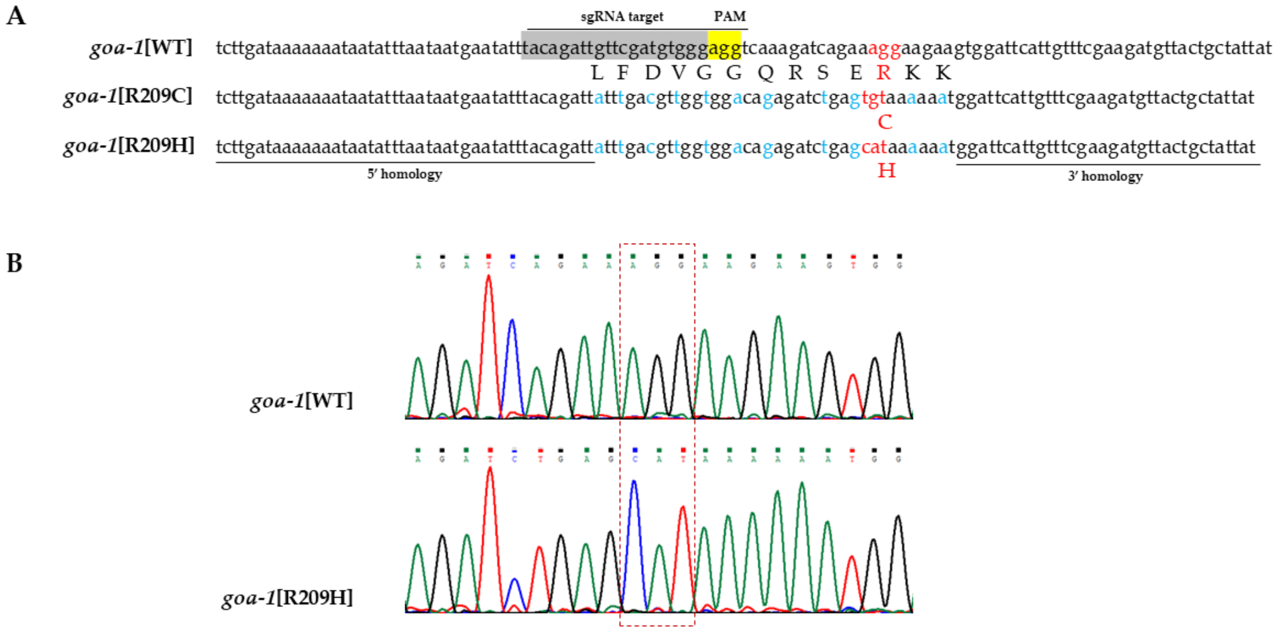

Figure S1. Gene editing strategy at codon 209 and Sanger sequencing validation. (**A**) Gene editing design through multiplexed CRISPR/Cas9 for R209C and R209H mutations. Gene edits and resulting amino acid substitutions are highlighted in red. Silent changes that did not alter the coding sequence, introduced to prevent Cas9 re-cutting and to ease PCR-based genotyping are highlighted in cyan (**B**) Chromatograms showing the presence of the R209H mutation.

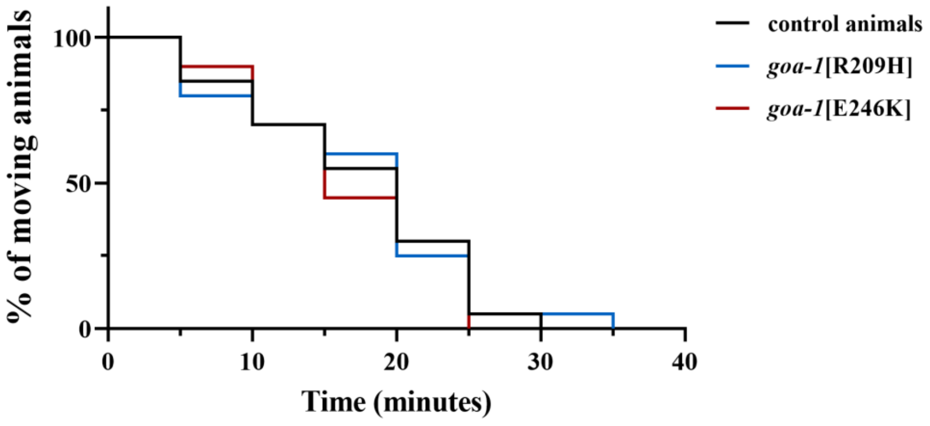

Figure S2. Mutant animals show normal sensitivity to levamisole. *goa-1*[R209H] and *goa-1*[E246K] animals display normal sensitivity to levamisole-induced paralysis (1 mM), confirming the presynaptic origin of the cholinergic defect. Twenty animals for each genotype were analyzed.

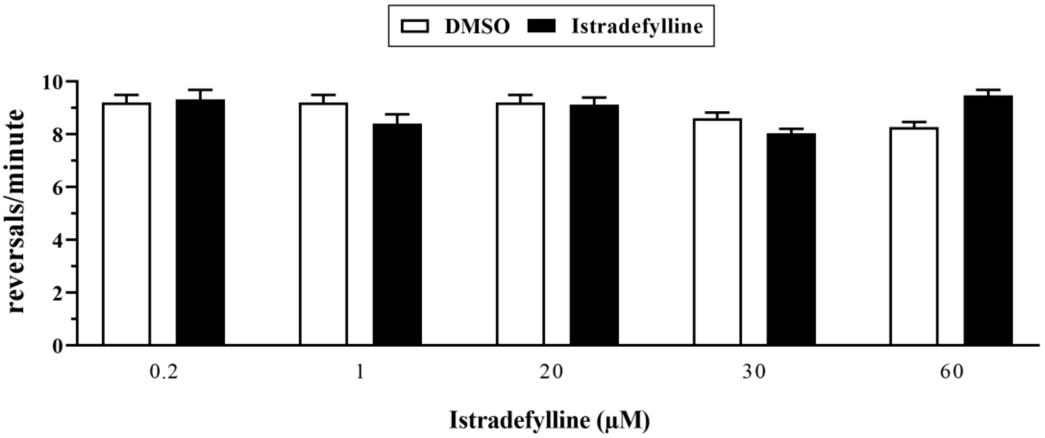

Figure S3. Istradefylline is not effective in *goa-1*[E246K] animals. Exposure to different concentrations of istradefylline (2 hours) did not induce a significant reduction in the number of reversals per minute in *goa-1*[E246K] animals. Twenty animals of each genotype were tested. Data represent mean ± SEM of multiple observations.
